# Supplementary figures and images for: STAT3 expression is reduced in cardiac pericytes in HFpEF and its loss reduces cellular adhesion and induces pericyte senescence
Source: FEBS Lett. 2025 May 1;599(12):1781–94. doi: 10.1002/1873-3468.70057 (PMC12183626; doi:10.1002/1873-3468.70057)

A

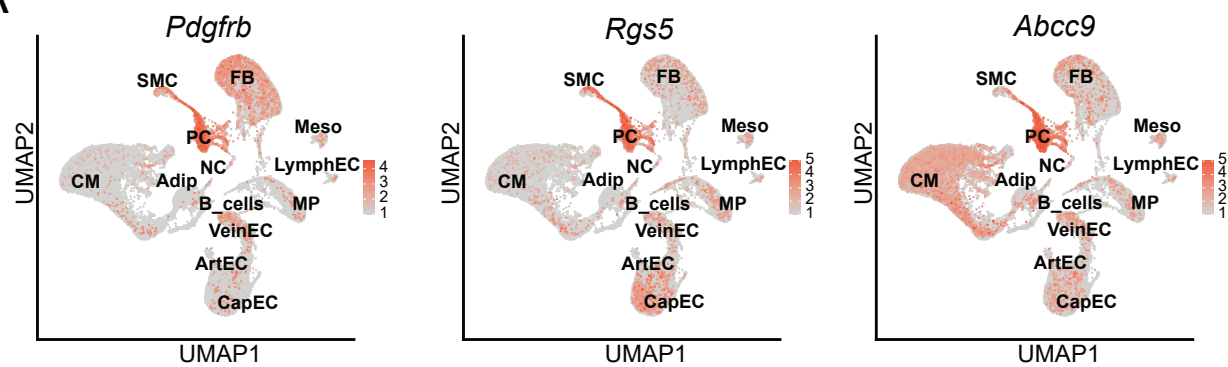

B

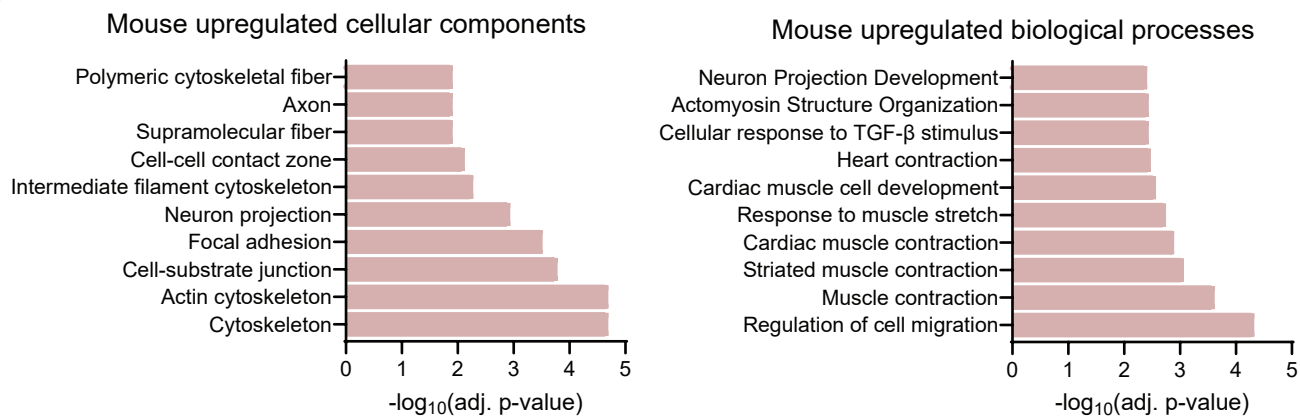

Supplement: Supplementary file 1 — Fig. S1. Gene Ontology (GO) enrichment analysis of significant differentially expressed genes. [file FEB2-599-1781-s003.pdf]

**A**

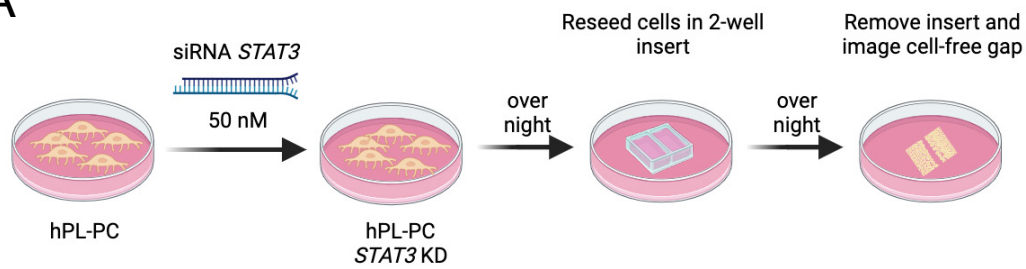

**B**

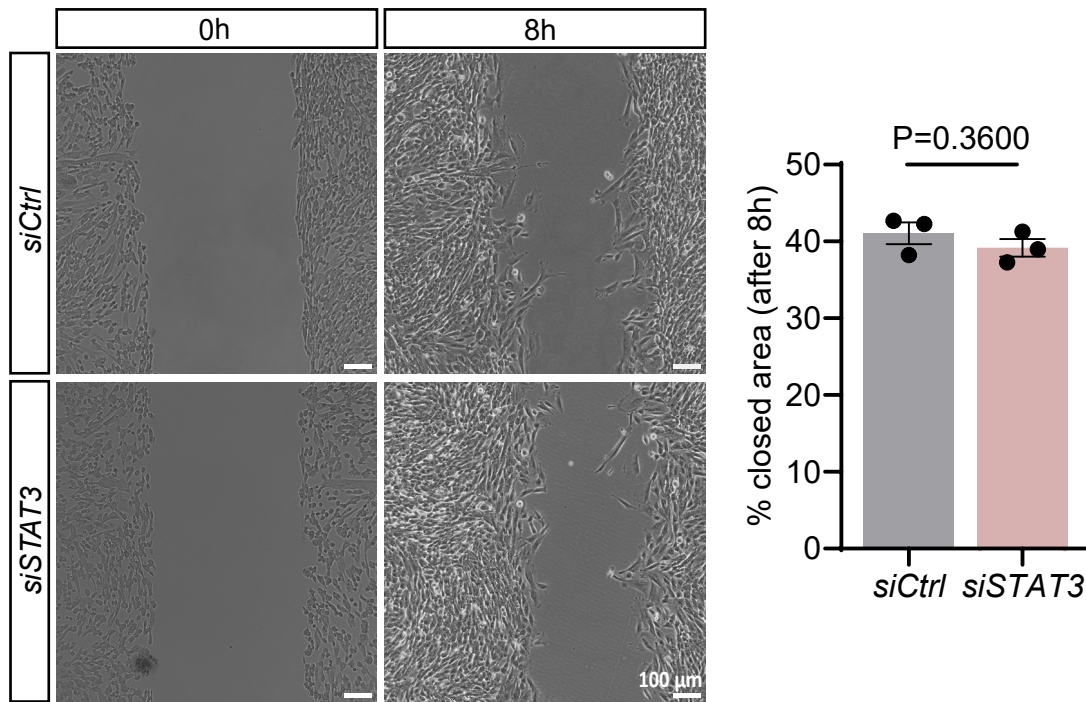

**C**

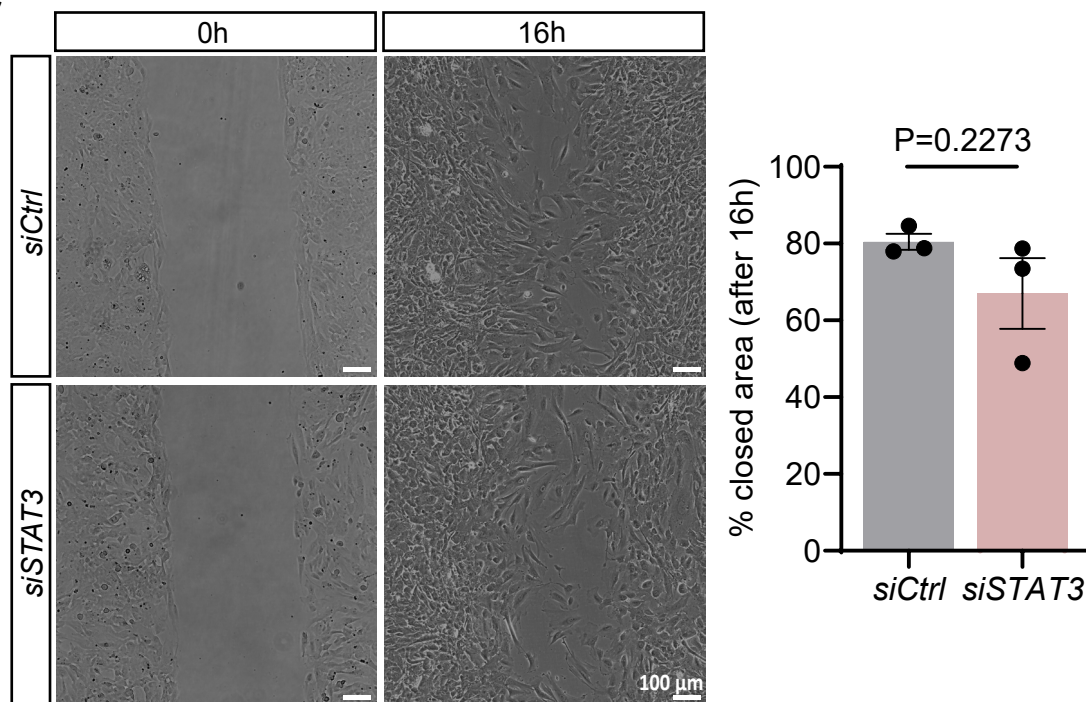

Supplement: Supplementary file 2 — Fig. S2. STAT3 deficiency does not significantly affect cellular migration. [file FEB2-599-1781-s001.pdf]

A

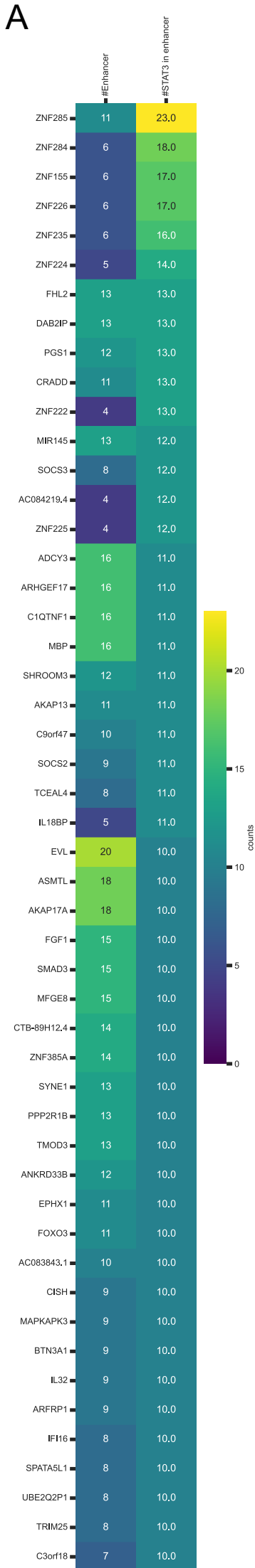

B

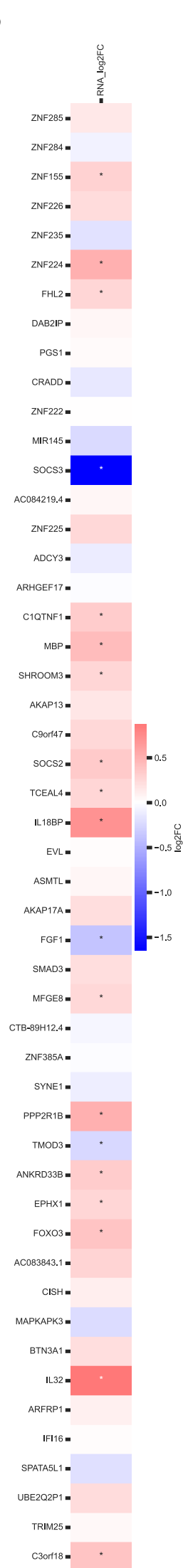

Supplement: Supplementary file 3 — Fig. S3. Integrated analysis of the DEGs using epigenome data and STAT3 motif matches. [file FEB2-599-1781-s002.pdf]
